# A phase I and pharmacokinetic study of novel taxane BMS-188797 and cisplatin in patients with advanced solid tumours

**DOI:** 10.1038/sj.bjc.6602886

**Published:** 2005-12-06

**Authors:** A du Bois, B Jung, A Loehr, T Schaller-Kranz, M Cohen, N Frickhofen

**Affiliations:** 1 Department of Gynaecology and Gynaecologic Oncology, HSK, Dr Horst Schmidt Klinik, Ludwig-Erhard-Str. 100, Wiesbaden D-65199, Germany; 2 Department of Hematology/Oncology, HSK, Dr Horst Schmidt Klinik, Wiesbaden D-65199, Germany; 3 Bristol-Myers Squibb GmbH, München 80809, Germany; 4 Bristol-Myers Squibb, Princeton, NJ 08453, USA

**Keywords:** BMS-188797, cisplatin, taxane

## Abstract

This phase I study investigated the maximum tolerated dose and pharmacokinetics of a 3-weekly administration of BMS-188797, a paclitaxel derivate, at three dose levels (DLs) (80, 110 and 150 mg m^−2^ DL), combined with cisplatin (standard dose 75 mg m^−2^). In 16 patients with advanced malignancies treated, one patient experienced dose-limiting febrile neutropenia, sepsis and severe colitis at the 150 mg m^−2^ DL; at the 110 mg m^−2^ DL one episode of dose-limiting grade 3 diarrhoea/nausea occurred. Grade 3/4 haematological toxicities were leucopenia/neutropenia; grade 3 nonhaematological toxicities were neuropathy, nausea, diarrhoea and stomatits. Objective response was seen in four patients, with three complete remissions in ovarian and cervical cancer patients. Pharmacokinetics of BMS-188797 appeared linear through the 110 mg m^−2^, but not through the 150 mg m^−2^ DL. The mean±SD values for clearance, distribution volume at steady state and terminal half-life during cycle 1 were 317±60 ml min^−1^ m^−2^, 258±96 l m^−2^ and 30.8±7.7 h, respectively. The maximum tolerated and recommended phase II dose for BMS-188797 was 110 mg m^−2^ (1-h infusion, every 3 weeks) combined with cisplatin 75 mg m^−2^.

Paclitaxel was the first member of a novel class of cytotoxics, the taxanes, that stabilise microtubules, causing cell cycle arrest and apoptosis (Rowinsky *et al*, 1993). The approved taxanes, paclitaxel and docetaxel, have a broad spectrum of antitumour activity both as single agents and in combination, and are widely used for cancer treatment (Choy, 2001). The clinical use of these agents, however, is limited by drug resistance and toxicities which include myelosuppression and peripheral neuropathy (Rowinsky *et al*, 1993). Therefore paclitaxel derivates have been synthesised, with the goal of achieving a broader spectrum of antitumour activity and a more favourable toxicity profile (Rose *et al*, 2001).

BMS-188797 is a novel, second-generation taxane which possesses a single structural modification from paclitaxel at the C-4 position to form the 4-desacetyl-4-methyl carbonate derivate of paclitaxel. BMS-188797, like paclitaxel, causes G2/M cell cycle arrest and exhibits potent antiproliferative activity against human tumour cell lines highly resistant to paclitaxel, either from overexpression of P-glycoprotein or because of specific mutations in beta-tubulin. This could be confirmed by the potent *in vivo* activity reported for BMS-188797 in paclitaxel-resistant tumour models in mice (Rose *et al*, 2001). A phase I study of BMS-188797 (1-h infusion given every 3 weeks) showed a maximum tolerated dose (MTD) of 175 mg m^−2^ (Sullivan *et al*, 2000).

Paclitaxel has been successfully combined with many clinically noncross-resistant agents, including the platinums, the latter especially in ovarian cancer (Neijt *et al*, 2000; Piccart *et al*, 2000; du Bois *et al*, 2003), breast cancer (Rosati *et al*, 2000), and non-small-cell lung cancer (NSCLC) (Smit *et al*, 2003). Consequently, the combination of platinum and BMS-188797 was evaluated. The first phase I combination study reported an MTD of 125 mg m^−2^ BMS-188797 with carboplatin AUC 5, every 3 weeks (Sullivan *et al*, 2001). Dose-limiting toxicities (DLT) were febrile neutropenia and slow recovery from neutropenia. Therefore, further effort was undertaken to develop a combination utilising the less myelosuppressive platinum analogue cisplatin.

Here we report a phase I study evaluating the combination of escalating doses of BMS-188797 as a 1-h infusion followed by cisplatin at a standard dose of 75 mg m^−2^ given every 3 weeks in patients with advanced solid tumours.

## PATIENTS AND METHODS

### Eligibility criteria

The following inclusion criteria had to be met: Written informed consent, histologically/cytologically confirmed malignant tumour after failure of standard therapy; measurable/nonmeasurable disease; adequate bone marrow function (absolute neutrophil count (ANC) ⩾2000 *μ*l^–1^; platelets ⩾100,000 *μ*l^–1^), hepatic function (bilirubin⩽1.5 mg dl^–1^; ALT/AST ⩽2.5 × ULN) and renal function (serum creatinine ⩽1.5 × ULN); age ⩾18 years; Eastern Cooperative Oncology Group (ECOG) performance status 0–2; no more than two prior chemotherapy regimens for metastatic disease and/or >2 prior (neo)adjuvant regimens. No taxane or platinum therapy was permitted within 4 months prior to study entry. Patients could not have brain metastases, pre-existing ototoxicity, pre-existing neuropathy of the National Cancer Institute Common Toxicity Criteria (NCI CTC, Version 2.0) grade ⩾1 or prior severe hypersensitivity reaction to agents containing polyoxyethylated castor oil (Cremophor EL).

### Study design and methodology

This study was an open-label, single-centre, phase I, dose-escalation trial with the primary objective to determine the MTD and DLT and to recommend a future phase II dose. Secondary objectives were safety, antitumour activity and pharmacokinetics of the experimental drug. The study was carried out with ethical committee approval.

### Treatment plan

BMS-188797 administered by a 1-h infusion was followed by a 1-h infusion of cisplatin starting 30 min after the end of the BMS-188797 infusion; both drugs were given on day 1, every 3 weeks. Six treatment cycles were planned. Treatment had to be discontinued upon progression or occurance of intolerable toxicity.

BMS-188797 was supplied in 50-mg vials containing 50% Cremophor EL and dehydrated ethanol. The drug was diluted before infusion with 0.9% NaCl or 5% dextrose to a final concentration of 0.3–1.2 mg ml^–1^ and administered through polyethylene-lined administration sets. Cisplatin solution was further diluted in 250 ml 0.9% NaCl. Escalating dose levels of 80, 110 and 150 mg m^−2^ BMS-188797 were administered to three, 12 and one patient, respectively. No intrapatient dose escalation was allowed for individual patients.

On day 1 of every cycle the antiemetic therapy with 5HT3-antagonists and antihypersensitivity premedication consisting of dexamethasone 20 mg, diphenhydramine 50 mg (or equivalent) and cimetidine 300 mg or ranitidine 50 mg was administered before BMS-188797 administration. Prehydration consisting of 1000–1500 ml 0.9% saline administered over 90 min was started at the same time as the initiation of the 1-h BMS-188797 infusion. At the end of the BMS-188797 infusion, 250 ml mannitol were administered over 30 min. After prehydration and mannitol infusion, the cisplatin infusion was administered over 1 h. Upon completion of the cisplatin infusion, posthydration consisting of 1500 ml 0.9% saline was administered over 3 h. Antiemetic prophylaxis for delayed emesis was given on days 2–5. Haematopoetic growth factors were not administered prophylactically.

For retreatment on day 22, patients had to have an ANC ⩾1500 *μ*l^−1^ and a platelet count ⩾100,000 *μ*l^–1^; with all treatment-related toxicities (except alopecia) recovered to baseline or to CTC grade ⩽1. If a patient was unable to meet retreatment criteria on day 22, treatment was delayed for 1 week for up to 3 weeks. Any delay >21 days resulted in removal from the study.

### Dose-limiting toxicities and MTD

Three patients were treated at each dose level (DL) prior to dose escalation. If one DLT was observed during the first course of therapy in one of these three patients, then three additional patients were treated. If no further DLT occurred, the next DL was opened. If the total number of patients with DLT at any DL was two or higher, then dose escalation was terminated and additional patients were enrolled at the next lower DL. The MTD was defined as the DL at which none of the three or one of six patients experienced a DLT. Once the MTD had been established, accrual at the respective DL was expanded to determine its suitability as the recommended phase II dose.

The DLT was defined as first-course toxicity with ANC <500 *μ*l^−1^ for ⩾5 days, or febrile neutropenia (fever >38.5°C and ANC <1000 *μ*l^−1^), or thrombocytopenia <25,000 *μ*l^–1^; or bleeding requiring platelet transfusion, or any other drug-related grade ⩾3 toxicity except fatigue/asthenia, transient arthralgia/myalgia, or grade 3 AST/ALT elevation (resolving to baseline within 3 weeks). Furthermore, delayed recovery from toxicity related to treatment with BMS-188797 and cisplatin that delayed scheduled retreatment for 21 days or longer was regarded as DLT.

### Dose modifications

Patients experiencing DLT (excluding grade 3 neuropathy) could be retreated but with a reduced dose in subsequent courses. No more than two dose reductions were permitted. No dose re-escalation was allowed. Toxicities requiring a BMS-188797 dose reduction by one DL were: ANC <500 *μ*l^–1^ for ⩾5 days, febrile neutropenia, platelets <25,000 *μ*l^–1^, grade ⩾3 thrombocytopenia with bleeding requiring transfusion, grade ⩾3 diarrhoea, and grade 2 neuropathy. Toxicities resulting in decreased cisplatin dose (50 mg m^−2^) were grade ⩾3 nausea/vomiting (despite medical intervention) and grade 2 neuropathy. Toxicities resulting in treatment discontinuation were recurrent grade ⩾3 nausea/vomiting and grade 2 neuropathy despite dose reduction, grade ⩾3 neuropathy, inner ear/hearing, toxicity, and elevated creatinine.

### Toxicity and response evaluation

Toxicity was evaluated according to NCI CTC (Vers. 2.0; revised April 30, 1999) in all patients receiving study drug. Tumour measurement was performed in patients with measurable disease after every other cycle; response was assessed according to World Health Organization (WHO) criteria. Ascites or serum tumour marker elevations were not considered in the assessment of response status except in case of progressive disease or complete remission when both had to be normalised. Patients evaluable for response had to have completed at least two courses of treatment or have disease progression.

### Sample collection and drug analysis

In all, 5 ml of blood were collected for pharmacokinectic analysis using Becton-Dickenson Vacutainers® that contained K_3_EDTA as the anticoagulant. Blood sampling for pharmacokinetics analysis was performed in all patients during cycle 1. Serial blood samples were drawn at the following times relative to the start of the 1 h infusion of BMS-188797: predose, 30 min, 58 min (drawn prior to the end of the infusion), and 1.25, 1.5, 2, 2.5, 3, 4, 5, 6, 8, 24, 48 and 72 h. Within 1 h of collection, the plasma was separated by centrifugation at 1000 rpm for 15 min at 4°C. Plasma was stored at or below 20°C until analysis. Plasma samples were analysed for BMS-188797 concentrations by HPLC. After the addition of internal standard, BMS-183061, to 1.0 ml of plasma, the sample was loaded onto a CN-U solid-phase extraction column. The compounds were eluted with 0.1% formic acid in methanol, the eluate evaporated to dryness, and the residue reconstituted. Chromatographic separation of the compounds was achieved on a YMC-ODS-AQ, 4.6 × 150 mm, 3 *μ*m column using a mobile phase containing 30% water in acetonitrile. Detection was by ultraviolet absorbance at 228 nm. The standard curve range was 2–1000 ng ml^−1^. The coefficient of variation (CV) for the between- and within-run precision for analytical quality control samples were no greater than 3.0 and 7.1%, respectively

### Pharmacokinetic analysis

Estimates of pharmacokinetic parameters for BMS-188797 were derived from individual concentration–time data sets by noncompartmental analyses (Gibaldi and Perrier, 1982). The values of the maximum plasma concentration (*C*_max_) were recorded directly from experimental observations. The area under the plasma concentration vs. time curve from time zero to the time of the last measurable concentration *T* (AUC_0-*T*_) was calculated using a combination of linear and log trapezoidal summations. The first-order rate constant of decline of BMS-188797 concentrations in the terminal phase of the plasma concentration–time data set, *λ*, was estimated by log-linear regression, using no weighting factor, of at least three data points yielding a minimum mean square error. The absolute value of *λ* was used to estimate the apparent terminal elimination half-life, *t*_1/2_. The last measurable concentration and the rate constant, *λ*, were used to extrapolate the AUC_0-T_ to estimate AUC_0-∞_ (the area under the curve from time zero to infinity). The total body clearance (Cl) was calculated by dividing the dose by AUC_0-∞_. The volume of distribution at steady state (*V*_SS_) was calculated using standard noncompartmental methods.

## RESULTS

### Patient characteristics

A total of 16 patients entered the trial and patient characteristics are summarised in Table 1. Median age was 61 years (range, 43–69), and all but one patient had a good performance status (ECOG 0-1). Patients had a variety of diagnoses, with ovarian neoplasm being the most frequent (six of 16 patients), four patients had NSCLC, two patients suffered from cancer of unknown primary (CUP), and one patient each had ovarian sarcoma, cervical cancer, urachus carcinoma and breast cancer. All patients with ovarian cancer had been treated previously with chemotherapy, with one patient having received carboplatin/cyclophosphamide and five patients being treated with carboplatin/paclitaxel. Of these five patients, one had received further topotecan and carboplatin/epirubicin therapy and one had received second-line treosulfan. Prior irradiation had been administered to three patients with breast, cervical and lung cancer. Prior tamoxifen had been given to two patients, one with breast cancer after irradiation and one with ovarian cancer after having received three prior chemotherapy regimens. Seven patients with lung cancer (three), CUP (two), urachus carcinoma and ovarian sarcoma had not received any prior therapy at study entry.

### Dose escalation

Dose escalation for BMS-188797 started at DLDL 1 80 mg m^−2^, which, at the time of study commencement, was shown to be safe and active from previous phase I trials. Cisplatin was administered at a fixed standard dose of 75 mg m^−2^. At the 80-mg m^−2^ DL, three patients received 19 courses. A total of 12 patients received 48 cycles at DL 2 (110 mg m^−2^ BMS-188797) and one patient received two cycles at DL 3 (150 mg m^−2^ BMS-188797).

### Dose-limiting toxicities and MTD

None of the three patients treated at DL 1 (80 mg m^−2^ BMS-188797) experienced a DLT. Consequently, the dose of BMS-188797 was escalated to 110 mg m^−2^. The second patient entered in this cohort experienced grade 3 diarrhoea and nausea. She received i.v. fluids, loperamide, dexamethson and metoclopramide and recovered within 4 days. According to protocol, a total of six patients had to be accrued at the same DL. None of the additional patients experienced a DLT, so the study could proceed to 150 mg m^−2^. The first patient entered in this cohort experienced a very severe episode of febrile neutropenia with e.coli-sepsis (SIRS criteria) and near fatal colitis, requiring i.v. treatment with fresh frozen plasma, ATIII, i.v. antibiotics and parenteral nutrition during the second course. Although according to the protocol DLTs were predefined toxicities occurring during course 1 only, it was decided to consider this toxicity a DLT due to its severity. Considering this nearly fatal adverse event combined with the ongoing observation that at the 110 mg m^−2^ DL, neutropenia was already increasing in severity over subsequent courses, we decided to prematurely close the 150 mg m^−2^ DL and stop further dose escalation of BMS-188797. Therefore 110 mg m^−2^ was considered the MTD of BMS-188797 in combination with cisplatin (75 mg m^−2^). According to protocol, six additional patients were enrolled at the expanded 110 mg m^−2^ DL. Since no further DLTs occurred, 110 mg m^−2^ BMS-188797 in combination with cisplatin (75 mg m^−2^), given as a 1-h infusion every 3 weeks was defined as the recommended dose for future phase II trials. Data on DLTs and MTD are summarised in Table 2.

### Toxicities

Grade 3/4 haematologic toxicities were observed with leukocytopenia and neutropenia at every DL investigated. The median time to the neutrophil nadir was 12, 14 and 8 days in DL 1, DL 2 and DL 3, respectively. The median duration of neutropenia was 4, 8 and 4 days at the respective DLs. Grade 4 neutropenia was observed in four out of 12 patients at the 110 mg m^−2^ dose level. Out of these four patients, two experienced grade 4 neutropenia for more than 5 days, which would have been defined as DLT if occurring during the first treatment cycle. As described above, this observation of prolonged neutropenia, increasing in severity at DL 2, confirmed our decision to terminate further dose escalation and expand accrual to the 110 mg m^−2^ DL for further phase II recommendation. Grade 4 neutropenia and febrile neutropenia resulting in sepsis was observed in the first patient entering DL 3 (150 mg m^−2^). The same patient experienced the only observed episode of grade 3 thrombocytopenia. No further grade 3/4 thrombocytopenia or anaemia was observed at any DL.

In this study, one grade 4 nonhaematologic toxicity, peritonitis without neutropenia, was observed. This toxicity, however, was not considered study drug related, but related to the underlying disease. No other grade 4 nonhaematologic toxicities occurred. Grade 3 nonhaematologic toxicities were observed in only one cycle per patient as follows: At the 80 mg m^−2^ DL, one patient, who had previously been treated with carboplatin and paclitaxel showed auditory/hearing toxicity. At the 110 mg m^−2^ DL grade 3 diarrhoea, nausea, infection without neutropenia, and fever occurred in one of 12 patients. Together with the haematologic toxicity observed at this DL, this DLT of grade 3 diarrhoea and nausea confirmed the designation of 110 mg m^−2^ as the MTD. Grade 3 sensory and motor neuropathy was observed in two patients at the 110 mg m^−2^ DL only. Neither patients had received prior taxane-containing chemotherapy and both experienced neuropathy in the last treatment cycle. One patient had sensory and motor neuropathy during cycle 4. Treatment was discontinued in this patient after cycle 4 due to disease progression. The second patient developed sensory neuropathy at cycle 6, just before treatment completion and after achieving a complete remission. Neuropathy was still present in this patient 4 months after the last dose of study drug. Toxicity experienced by the only patient entering the 150 mg m^−2^ DL is described above.

All patients but one experienced alopecia with the earliest onset at cycle 1 and the latest at cycle 6. Prophylactic premedication with steroids and H1/H2-antagonists was administered to all patients. Mild hypersensitivity reactions, grade 2 rash and dyspnea, were observed in only one patient in two subsequent cycles at the 110-mg m^−2^ DL, leading to temporary treatment interruption (for 24 and 1 h, respectively). Four additional courses were given without any further hypersensitivity reaction observed, but these courses were administered at a reduced dose of BMS-188797 (80 mg m^−2^) due to grade 4 neutropenia.

### Dose delay and treatment duration

In this trial only two cycles had to be delayed, one for logistical reasons and one due to urinary infection One patient experienced a mild hypersensitivity reaction during the second and third cycle, so treatment was interrupted temporarily for 24 and 1 h, respectively, and then resumed. Dose reduction of BMS-188797 was necessary in two patients at DL 2 (110 mg m^−2^). One patient experienced DLT (grade 3 nausea and diarrhoea) and one patient had grade 4 neutropenia lasting 7 days. Since no re-escalation was foreseen, seven out of 69 courses were given at reduced doses of BMS-188797. Cisplatin was administered at the full dose (75 mg m^−2^) at every DL to all patients. A median of five cycles were administered per patient (range, 1–6) and treatment was completed as planned in seven patients. Treatment was discontinued because of disease progression in six patients, and two patients died within 30 days of their last therapy due to disease progression.

Two patients discontinued treatment due to adverse side effects (one patient had grade 3 hearing loss and the other had grade 3 nausea, grade 2 vomiting, and fatigue).

### Antitumor activity

At DL 1, three patients with ovarian cancer, NSCLC, and CUP achieved stable disease as their best response. At DL 2 (BMS-188797 110 mg m^−2^) three complete remissions were observed in patients with ovarian (two patients) and cervical cancer (one). One partial response was observed in another patient with ovarian cancer. Stable disease was observed in two patients with urachus carcinoma and NSCLC. Four patients with lung cancer (two patients), ovarian sarcoma, and CUP had progressive disease. At DL 3 (BMS-188797 150 mg m^−2^) the only patient treated who was suffering from CUP had disease progression.

### Pharmacokinetic analyses

**Figure 1:**
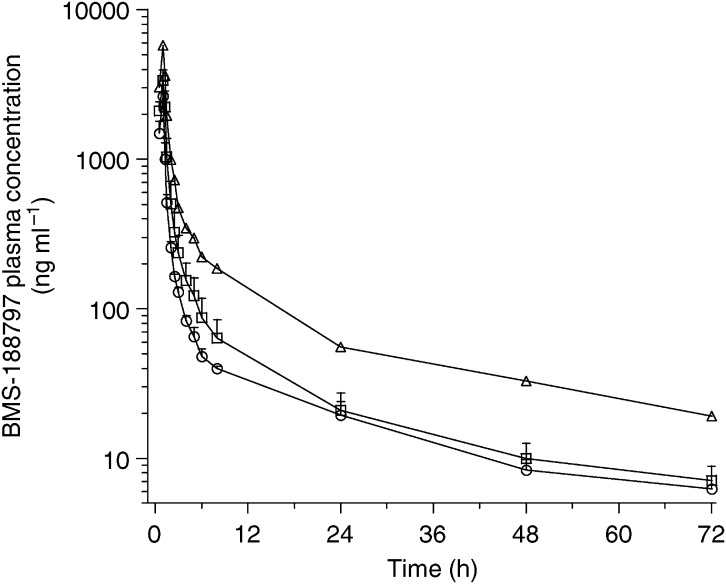
Mean BMS-188797 concentration–time profiles after administration of BMS-188797 80 mg m^−2^, ○; 110 mg m^−2^, □; 150 mg m^−2^, ▵; in combination with 75 mg m^−2^ of cisplatin. Error bars indicate s.d.

**Figure 2:**
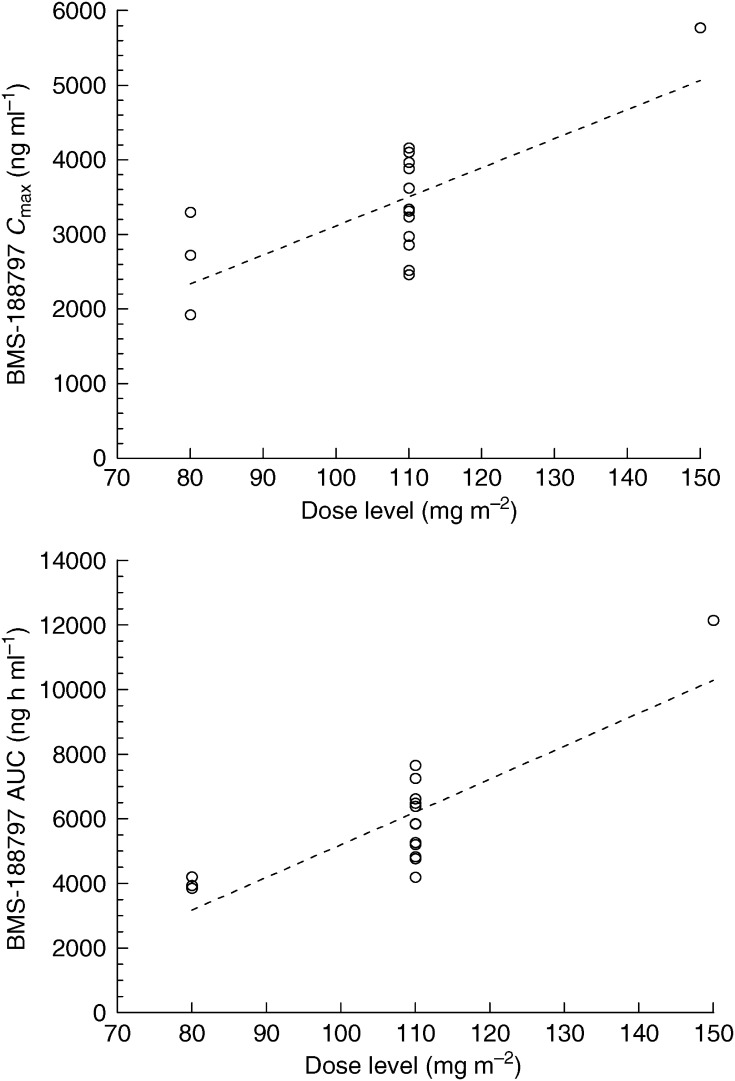
Individual BMS-188797 *C*_max_ and AUC values, ○; fit of the data derived from linear least squares regression, - - -.

Evaluable plasma concentration–time profiles were obtained from all 16 patients during the first cycle of treatment. Plasma concentrations were quantifiable in all patients through 72 h. Mean plasma concentration–time profiles of BMS-188797 are shown in Figure 1 and mean BMS-188797 pharmacokinetic parameters are listed in Table 3. The pharmacokinetics of BMS-188797 appeared independent of dose through the 110 mg m^−2^ DL, but not through the 150 mg m^−2^ DL. The mean±s.d. values for clearance, volume of distribution at steady state and terminal half-life of the three dose groups during cycle 1 were 317±60 ml min^−1^ m^−2^, 258±96 l m^−2^ and 30.8±7.7 h, respectively. At the recommended phase II dose of 110 mg m^−2^, interpatient variability in the principal pharmacokinetic parameters was moderate, with coefficients of variation percentages of 19, 35 and 24 for CL, *V*_SS_ and *t*_1/2_, respectively. At the 110 mg m^−2^ DL, there was a 1.8-fold range of AUC_0-∞_ values. The relationship of individual values of *C*_max_ and AUC_0-∞_ to dose are plotted in Figure 2. There was overlap in *C*_max_ and AUC_0-∞_ values among the 80 and 110 mg m^−2^ doses.

## DISCUSSION

Taxanes show a broad spectrum of antitumour activity and are active both as single agents or in combination with other noncross-resistant agents, including the platinums (Choy, 2001). Despite the progress that had been made using taxanes in ovarian (Neijt *et al*, 2000; Piccart *et al*, 2000; du Bois *et al*, 2003), breast (Rosati *et al*, 2000) and lung cancer (Smit *et al*, 2003), the majority of patients developed secondary drug resistance and, eventually, disease progression. Hence the taxane derivatives BMS-188797 and BMS-184476 were developed, with the aim of reducing toxicity and a broader spectrum of antitumour activity (Rose *et al*, 2001). Phase I single-agent studies have investigated BMS-188797 administered in 3-weekly (Sullivan *et al*, 2000) and weekly treatment intervals (Goldstein *et al*, 2001; Advani *et al*, 2003). The final report by Advani *et al* (2003) suggested an MTD of BMS-188797 of 50 mg m^−2^ for weekly administration. For the 3-weekly treatment schedule, an MTD of 175 mg m^−2^ was determined (Sullivan *et al*, 2000). At the start of this phase I combination study of BMS-188797 and cisplatin, the phase I single-agent trial investigating 3-weekly treatment intervals was still actively recruiting patients at the 175 mg m^−2^ DL, and the interim analysis suggested that BMS-188797 80–100 mg m^−2^ would be safe for subsequent combination studies.

Since the combination of taxanes and platinum compounds have shown a broad spectrum of antitumour activity, phase I studies investigating weekly and 3-weekly treatment intervals of BMS-188797 in combination with carboplatin administered every 3 weeks were initiated. Preliminary data of weekly BMS-188797 administration with carboplatin reported DL, prolonged grade 4 neutropenia and delayed haematological recovery at DLs AUC 6/33 mg m^−2^ and AUC 5/43 mg m^−2^ (Advani *et al*, 2002). The phase I study investigating the 3-weekly administration of both drugs reported febrile neutropenia and slow recovery from neutropenia to be DL. The MTD was carboplatin AUC 5/BMS-188797 125 mg m^−2^ (Sullivan *et al*, 2001). In summary, all BMS-188797 phase I studies reported so far have shown neutropenia to be the main DLT. Therefore, this phase I study was undertaken to investigate the combination of BMS-188797 with the potentially less myelosuppressive platinum analogue cisplatin. However, the study failed to show that the combination of standard-dose cisplatin (75 mg m^−2^) with BMS-188797 was less myelosuppressive. Dose-limiting toxicities were observed from DL 2 on (BMS-188797 110 mg m^−2^), and further dose escalation could not be achieved. Of the 12 patients treated at the MTD level of BMS-188797 110 mg m^−2^, two needed dose reduction for grade 3 diarrheoa/nausea and for long-lasting neutropenia, respectively, and treatment cycles had to be delayed in one additional patient for urinary tract infection. At this DL, another three patients experienced prolonged and increasingly severe neutropenia over the course of treatment. It was therefore unlikely that further dose escalation to the 150 mg m^2^ DL could be safely accomplished and our earlier decision to prematurely close the 150-mg m^−2^ DL was confirmed. Thus, the MTD was determined to be BMS-188797 110 mg m^−2^ in combination with cisplatin 75 mg m^−2^, given every 3 weeks. This dose was slightly lower than the 125-mg m^−2^ dose of BMS-188797 achieved in combination with carboplatin AUC 5 in the study reported by Sullivan *et al* (2001). Similarly, a better safety profile has also been demonstrated for carboplatin/paclitaxel combinations in patients with ovarian cancer when compared to cisplatin/paclitaxel (du Bois *et al*, 2003). The observed toxicity profile of cisplatin/BMS-188797 was rather similar to cisplatin/paclitaxel and the phase I trial presented here could not show an improved safety profile. This comparison, however, can only be performed on a qualitative basis, considering the early phase of clinical development and the limited number of patients treated with BMS-188797/cisplatin. However, activity was observed at the MTD of BMS-188797/cisplatin in several tumours, which needs further evaluation. Although no conclusions can be drawn from this small sample size, larger phase II studies could be planned to gain further evidence of activity and to support the decision to compare this new taxane analogue plus a platinum compound to paclitaxel plus a platinum drug.

The overall pharmacokinetics of BMS-188797 combined with cisplatin in this study were similar to those of BMS-188797 as a single agent and were characterised by a large volume of distribution and a long apparent terminal elimination half-life (Advani *et al*, 2003). However, the mean exposure in patients was lower in this study than in the single-agent studies or in a study of BMS-188797 in combination with carboplatin (Advani *et al*, 2003). Cisplatin would not be expected to increase the clearance of BMS-188797. However, one of the limitations of this study was that we did not look for interactions between both compounds and did not measure platinum pharmakokinetics. The pharmacokinetics of BMS-188797 in combination with cisplatin in this study appeared linear through a DL of 110 mg m^−2^. Firm conclusion for higher doses coud not be drawn due to the fact that only one patient was treated with 150 mg m^−2^. The clearance in this patient was approximately one-third lower than that in patients at 80 or 110 mg m^−2^ which might have attributed to the serious course of toxicity.

Another candidate for further trials might be the second novel paclitaxel derivative, BMS-184476, a 7-methylthiomethyl ether of paclitaxel, which was also investigated in single-agent phase I trials (Plummer *et al*, 2002) and in combination with cisplatin (Sun *et al*, 2003) and carboplatin (Bilenker *et al*, 2004). Similar to our results, neutropenia and diarrhoea were the DLTs for BMS-184476 in combination with cisplatin; other toxicities were comparable with paclitaxel/cisplatin. The occurrence of diarrhoea, which was noted also in the single-agent trials with BMS-184476 and BMS-188797, is unusual for paclitaxel treatment and might confirm the *in vitro* findings suggesting that the paclitaxel analogues have the ability to circumvent the P-glycoprotein pump, which is highly expressed in the colon mucosa (Thiebaut *et al*, 1987). It may be of interest to test these agents in tumours in which resistance is specifically associated with the multidrug-resistance phenotype.

In conclusion, our phase I trial showed that 3-weekly administration of BMS-188797 110 mg m^−2^ in combination with cisplatin 75 mg m^−2^ is active and safe and is recommended for further phase II trials. These initial findings warrant more extensive investigation into the activity of BMS-188797/cisplatin, particularly in patients with platinum-sensitive tumours not responding to paclitaxel.

## Figures and Tables

**Table 1:** Patient characteristics

	**Number of patients (%) (*n*=16)**
Age (years) – median (range)	61 (43–69)
	
*Performance status*	
0	11 (69)
1	4 (25)
2	1 (6 [TSK1])
	
*Sex*	
Male	5 (31)
Female	11 (69)
	
*Prior therapy*	
Radiotherapy	3 (19)
Chemotherapy only	6 (38)
No prior therapy	7 (44)
	
*Tumour type*	
Ovarian carcinoma	6
Ovarian sarcoma	1
Non-small-cell lung cancer	4
Cancer of unknown primary	2
Carcinoma of the cervix	1
Urachus carcinoma	1
Breast cancer	1

**Table 2:** Dose-limiting toxicities (DLTs) and maximum tolerated dose

**Dose level**	**BMS-188797**	**Cisplatin**	**No. of patients enrolled**	**No. of cycles administered**	**DLT observed in *n* patients**	**MTD**
DL 1	80 mg m^−2^	75 mg m^−2^	3	19	—	—
DL 2	**110 mg m^−2^**	75 mg m^−2^	12	48	Diarrhoea G3 (1 patient)	**MTD**
DL 3	150 mg m^−2^	75 mg m^−2^	1	2	Neutropenia G4, febrile neutropenia, sepsis, colitis G3 (one patient^a^)	—

a Toxicity was observed in second treatment cycle, but was considered DLT due to its life-threatening nature. MTD-level printed in bold.

**Table 3:** Pharmacokinetic parameters of BMS-188797 in patients

**Dose group (mg m−2)**	**No. of patients**	*****C*_max_** (ng ml−1)**	**AUC0-∞ (h ng ml−1)**	**Cl (ml min−1 m−2)**	*****t***1/2 (h)**	*****V*_SS_** (l m−2)**
80	3	2650±690 (1925, 3300)	4001±184 (3856, 4208)	333±14 (317, 344)	31.0±11.7 (18.1, 40.8)	331±135 (214, 478)
						
110	12	3371±591 (2462, 4162)	5868±1055 (4195, 7665)	322±60 (234, 437)	30.6±7.5 (14.4, 42.2)	244±86 (115, 395)
						
150	1	5773 NA	12155 NA	206 NA	31.4 NA	209 NA

NA=not applicable.

Data are means±s.d. with ranges in parentheses.
